# Shifting Dynamics of Dengue Virus Serotype 2 and Emergence of Cosmopolitan Genotype, Costa Rica, 2024

**DOI:** 10.3201/eid3111.250746

**Published:** 2025-11

**Authors:** Mauricio González-Elizondo, Dihala Picado Soto, Estela Cordero Laurent, Francisco Duarte Martínez, Luiz Carlos Junior Alcantara, Vagner Fonseca, Jairo Andrés Méndez Rico, Jose Lourenco, Leticia Franco, Marta Giovanetti, Claudio Soto Garita

**Affiliations:** Centro Nacional de Referencia de Virología, Tres Ríos, Costa Rica (M. González-Elizondo, D. Picado Soto, E. Cordero Laurent, F. Duarte Martínez, C. Soto Garita); René Rachou Institute, Oswaldo Cruz Foundation, Rio De Janeiro, Brazil (L.C.J. Alcantara); Universidade Federal de Minas Gerais Instituto de Ciencias Biologicas, Belo Horizonte, Brazil (L.C.J. Alcantara); University of the State of Bahia, Salvador, Brazil (V. Fonseca); Centre for Epidemic Response and Innovation (CERI), School of Data Science and Computational Thinking, Stellenbosch University, Stellenbosch, South Africa (V. Fonseca); Pan American Health Organization/World Health Organization, Washington, DC, USA (J.A. Méndez Rico, L. Franco); Universidade Católica Portuguesa, Católica Medical School, Católica Biomedical Research Centre, Lisbon, Portugal (J. Lourenco); Università Campus Bio-Medico di Roma, Rome, Italy (M. Giovanetti); Oswaldo Cruz Institute, Oswaldo Cruz Foundation, Minas Gerais, Brazil (M. Giovanetti)

**Keywords:** Dengue virus, viruses, dengue, vector-borne infections, mosquitoborne infections, genomic monitoring, cosmopolitan genotype, eco-epidemiological modelling, Costa Rica

## Abstract

Dengue remains a major public health challenge. In Costa Rica, we implemented nationwide genomic surveillance to track dengue virus serotype 2 cosmopolitan genotype emergence. Phylogenetic and eco-epidemiologic analyses revealed early detection, climate-driven spread, and spatial heterogeneity. Our findings underscore the need for integrated surveillance to guide adaptive responses to emerging arboviral threats.

Dengue fever, caused by mosquitoborne dengue virus (DENV), remains a major public health threat. DENV is primarily transmitted by *Aedes aegypti* mosquitoes (*1*). Rising global dengue incidence has been linked to climate change and urbanization (*2*). 

In Costa Rica, DENV transmission has become increasingly complex. Dengue cases rose from 30,649 in 2023 (*2*) to 31,259 in 2024 (*1*); San José, Alajuela, and Puntarenas reported the highest incidence rates. Inciensa launched a nationwide DENV sequencing program in 2023, which confirmed simultaneous circulation of all 4 serotypes (DENV-1–4). That study was approved by the Pan-American Health Organization Ethics Review Committee (reference no. PAHO-2024-08-0029) and was conducted as part of routine arbovirus surveillance at Inciensa. In February 2024, that surveillance detected DENV-2 genotype II (cosmopolitan); by September genotype II had fully replaced genotype III, and the earliest cases were reported in coastal districts (*3*,*4*). Genotype II is associated with more severe clinical outcomes (*4*) and has been reported in Peru and Brazil since 2019 (*3*,*4*), raising concerns for regional spread. We investigated whether ecologic factors were contributing to DENV shifts in Costa Rica.

## The Study

**Figure 1:**
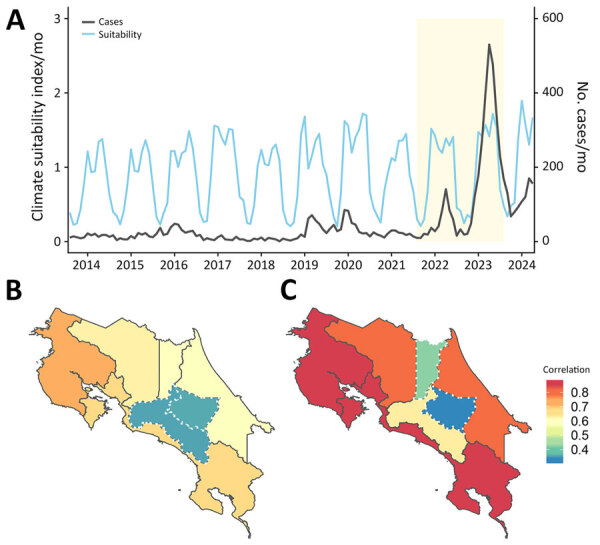
Temporal and spatial correlation between climate-driven suitability and dengue incidence from a study of shifting dynamics of dengue virus serotype 2 and emergence of cosmopolitan genotype, Costa Rica, 2024. A) Time series of monthly dengue cases and climate-driven suitability index for transmission during June 2014–November 2024. Shaded area (yellow) indicates the epidemic period during which enough cases with a clear seasonal signal were reported to enable an estimate correlation between suitability and incidence (Spearman r = 0.38; p<0.05). Scale bars for the y-axes differ substantially to underscore patterns. B, C) Province-level Spearman correlation values between monthly dengue incidence and climate suitability during 2022 (B) and 2023 (C). Warmer colors indicate stronger correlations. White dashed boundaries mark provinces with nonsignificant correlation (p>0.05) and solid dark gray boundaries indicate provinces with statistically significant correlation. In 2023, higher correlations were observed in eastern and coastal provinces where early cases of the dengue virus serotype 2 cosmopolitan genotype were detected.

To assess ecologic drivers, we compared dengue incidence with a climate-driven suitability index, which integrates temperature- and humidity-dependent mosquito traits, such as biting rate, lifespan, and extrinsic incubation. Before 2022, dengue activity was irregular in Costa Rica but surged during 2022–2024 (Figure 1, panel A); we noted a moderate correlation (r = 0.38) between suitability and incidence during the 2022–2023 epidemic (Appendix 1). At the province level, correlations in 2022 were broadly consistent (Figure 1, panel B), but in 2023, we observed stronger associations in Puntarenas and Limón, where the cosmopolitan genotype first appeared, suggesting ecologic and virologic factors converged to intensify local transmission (Figure 1, panel C).

Historically, DENV-1 and DENV-2 have been the predominant serotypes in Costa Rica, fluctuating in relative proportions. However, we observed a major shift in 2023–2024, characterized by co-circulation of all 4 serotypes, mirrored by emergence of DENV-4 in late 2022 and reemergence of DENV-3 in early 2023 after a 6-year absence (Appendix 1 Figure 1, panel A). The reemergence of DENV-3 aligns with the known ubiquitous serotype cycles observed every 7–9 years, and DENV-4 emergence aligns with its recent expansion in South America (*1*). 

To assess whether those serotype shifts were associated with longer-term changes in age distribution, we analyzed dengue case data spanning 2014–2024, the entire period of available national dengue surveillance. During the years with available dengue reports, age ranges among infected persons changed slightly, but we saw no quantifiably significant change over time (linear slope 0.17; p = 0.048) (Appendix 1 Figure 1, panel B). That estimate did not strongly support a substantial increase in the force-of-infection over time, which was supported by the relatively stable climate-driven suitability estimates (Figure 1). Force-of-infection should be mirrored by a decreasing age of reported infections; however, the age of infection increased slightly by 1.3 years for every extra circulating serotype (p<0.001), independent of year (Appendix 1 Figure 1, panel B). That finding potentially indicates that serotype mixing increased the prevalence for secondary infections, which then occurred in older persons who were already seropositive.

Concurrently, we observed a marked change in circulating DENV-2 strains; the previously dominant genotype III was replaced by genotype II in early 2024 (Appendix 1 Figure 1, panel C). During May 2023–August 2024, DENV-2 genotype III was more prevalent, particularly in Alajuela, San José, Puntarenas, and Limón, regions historically associated with high DENV transmission. Over time, however, genotype II became increasingly dominant, especially in San José, Cartago, and Alajuela, and genotype III declined. That pattern suggests a gradual replacement, potentially driven by selective advantage, immune escape, or repeated introductions from external sources.

**Figure 2:**
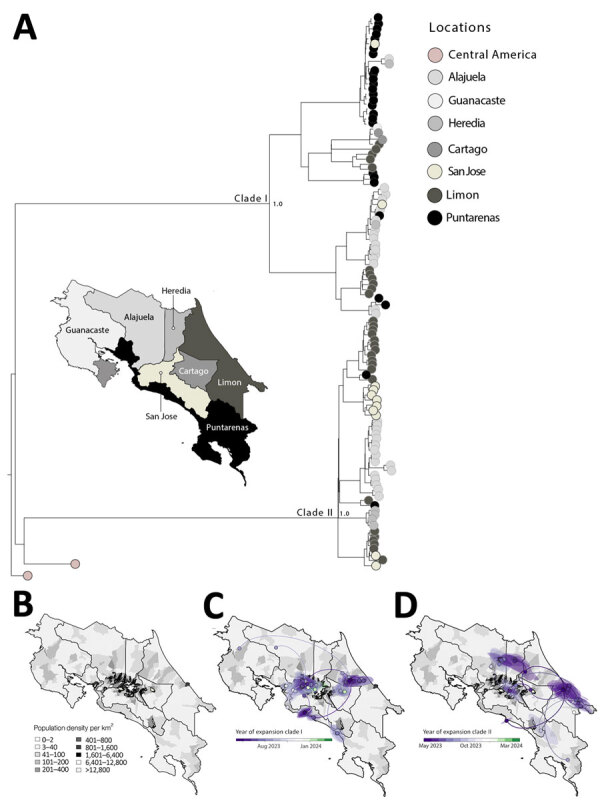
Maximum-likelihood phylogeny and spatiotemporal expansion of dengue virus (DENV) serotype 2 (DENV-2) and emergence of cosmopolitan genotype, Costa Rica, 2024. A) Time-stamped phylogenetic tree of DENV-2 genotype III, showing 2 independent introductions of this genotype into Costa Rica, including 1 major transmission cluster. Tips are colored according to sampling location. Scale bar indicates nucleotide substitutions per site. Full phylogeny shown in Appendix 1 Figure. B) Population density map of Costa Rica, highlighting areas with elevated DENV transmission potential. C, D) Spatiotemporal expansion of DENV-2 genotype III lineage D.1.2, illustrating the progressive spread of 2 distinct clades, clade I (C) and clade II (D), from urban centers to coastal regions between May 2023 and March 2024.

After launching a nationwide genomic surveillance program, Inciensa generated 133 DENV-2 whole-genome sequences during 2023–2024. Using the dynamic DENV lineage classification system (*5*), we assigned 110 genotype III (Asian–American) genomes to lineage D.1.2 and 23 genotype II (cosmopolitan) genomes to lineage F.1.1.2. Genotype III sequences were from 110 patients (mean age 38 years) across 7 provinces (Appendix 2 Table 1); mean genome coverage was 92.6%, and mean cycle threshold was 22. Genotype II sequences were from 23 patients (mean age 38 years) in 5 provinces (Appendix 2 Table 2); mean coverage was 80%, and mean cycle threshold was 24. Phylodynamic analyses showed a well-supported monophyletic clade of DENV-2 genotype III in Costa Rica (Figure 2, panel A; Appendix 1 Figure 2), consistent with sustained local persistence after introduction events from Central America over the previous decade.

Maximum-likelihood phylogenetic reconstruction revealed cocirculation of 2 distinct clades within the DENV-2 genotype III D.1.2 lineage, here referred to as clades I and II (Figure 2, panel B). Although phylogenetically distinct, both clades belong to the same lineage. Phylogeographic analysis showed that early circulation was concentrated in Alajuela, Cartago, and San José, followed by expansion toward the coastal regions of Puntarenas and Limón. Clade I likely emerged in May 2023 (95% highest posterior density [HPD] April–late May 2023) (Figure 2, panel D) and spread from San José and Cartago to Puntarenas and Limón by early 2024. Clade II (Figure 2, panel E), detected as early as June 2023 with a similar HPD, displayed broader dispersal, including to the densely populated areas of Alajuela and San José. The spatial overlap of those sublineages with high-population regions (Figure 2, panel C) underscores the role of urban centers as transmission hubs enabling spread of DENV-2. 

**Figure 3:**
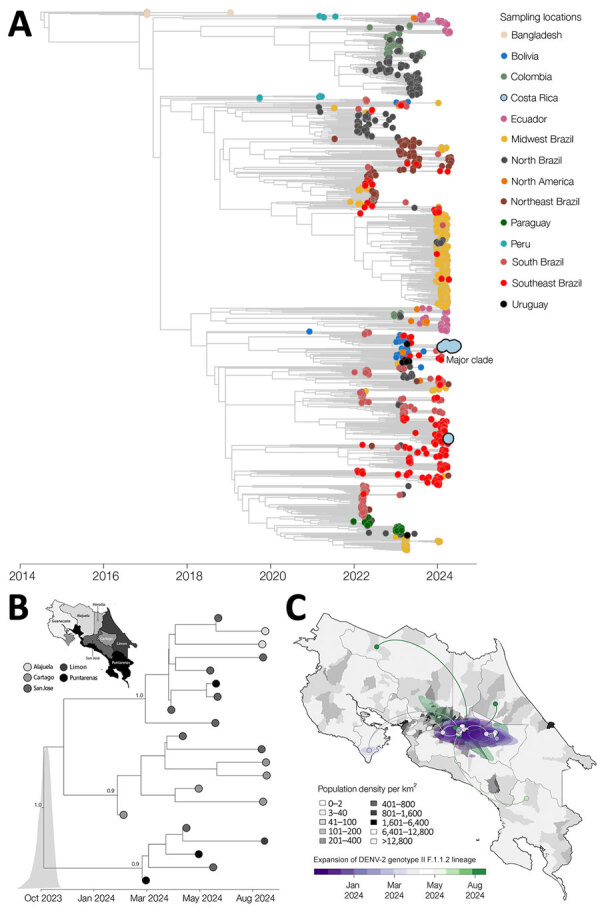
Time-scaled and spatiotemporal dynamics of dengue virus serotype 2 (DENV-2) and emergence of cosmopolitan genotype, Costa Rica, 2024. A) Time-scaled phylogenic reconstruction of global DENV-2 showing major clade in Costa Rica in 2024. Full phylogeny shown in Appendix 1 Figure. B) Time-scaled phylogenetic reconstruction of the major DENV-2 genotype II clade, illustrating its expansion from Puntarenas, Limón, and Cartago in early 2024 (map inset). C) Spatiotemporal dynamics of the major DENV-2 genotype II clade in Costa Rica demonstrating its spread among provinces. DENV, dengue virus.

Further phylogenetic resolution of DENV-2 genotype II sequences revealed a distinct evolutionary trajectory compared with DENV-2 genotype III (Figure 3, panel A; Appendix 1 Figure 3), supporting the hypothesis of recent introduction followed by rapid establishment in Costa Rica. The time-stamped phylogenetic tree indicated that >2 independent introductions of the DENV-2 genotype II F.1.1.2 lineage likely occurred, potentially mediated by regional viral flow from countries in Latin America, including Bolivia and Brazil, and resulted in establishment of a well-supported monophyletic clade. Bayesian time-scaled phylogenetic analysis of that clade suggests emergence around October 2023, with a 95% HPD interval spanning from September to late November 2023. Early circulation was primarily concentrated in Puntarenas, Limón, and Cartago, then subsequently disseminated into San José, Alajuela, and Heredia (Figure 3, panel B). Reconstruction of viral dispersal for that major clade further highlighted its rapid establishment across densely populated areas (Figure 3, panel C). Initially detected in coastal and central provinces, the virus quickly spread into high-transmission hubs, particularly those characterized by high population density.

## Conclusions

We used nationwide DENV genomic data and a suitability index to conduct an eco-epidemiologic assessment of dengue in Costa Rica. We documented replacement of DENV-2 Asian–American genotype by DENV-2 cosmopolitan genotype. Using sequencing, phylodynamics, and climate modeling, we showed how viral introductions, ecologic factors, and human mobility shaped transmission. Unlike other settings where genotype shifts were driven by immunity or fitness (*6*–*8*), we found no evidence of climate- or age-related increases. DENV-2 II, detected in early 2024, rapidly replaced DENV-2 genotype III despite declining circulation and did not show increased severity or deaths. At least 2 introduction events seeded widespread dissemination, consistent with patterns in Brazil and Southeast Asia (*9*,*10*). Now globally dominant, the cosmopolitan genotype has also been reported in Peru, Brazil, and Colombia (*3*,*4*,*11*). Its moderate correlation with climate suitability (r = 0.38) (*2*,*12*) and spread into urban centers (*13*–*15*) highlight how ecologic and mobility factors can amplify transmission. Those findings underscore the urgent need for real-time genomic surveillance integrated with environmental and mobility data to strengthen early dengue detection and targeted interventions.

Appendix 1 Additional information shifting dynamics of dengue virus serotype DENV-2 and emergence of cosmopolitan genotype, Costa Rica, 2024.

Appendix 2 Genomic data used in a study of shifting dynamics of dengue virus serotype DENV-2 and emergence of cosmopolitan genotype, Costa Rica, 2024.

## References

[1] Pan-American Health Organization (PAHO). Dengue epidemiological situation in the region of the Americas: epidemiological week 40, 2025 [cited 2025 Mar 22]. https://www.paho.org/en/documents/dengue-epidemiological-situation-region-americas-epidemiological-week-08-2025

[2] Nakase T, Giovanetti M, Obolski U, Lourenço J. Population at risk of dengue virus transmission has increased due to coupled climate factors and population growth. Commun Earth Environ. 2024;5:475. 10.1038/s43247-024-01639-6

[3] García MP, Padilla C, Figueroa D, Manrique C, Cabezas C. Emergence of the Cosmopolitan genotype of dengue virus serotype 2 (DENV2) in Madre de Dios, Peru, 2019. Rev Peru Med Exp Salud Publica. 2022;39:126–8. 10.17843/rpmesp.2022.391.10861 35766734 PMC11397596

[4] Giovanetti M, Pereira LA, Santiago GA, Fonseca V, Mendoza MPG, de Oliveira C, et al. Emergence of dengue virus serotype 2 cosmopolitan genotype, Brazil. Emerg Infect Dis. 2022;28:1725–7. 10.3201/eid2808.220550 35876608 PMC9328905

[5] Hill V, Cleemput S, Pereira JS, Gifford RJ, Fonseca V, Tegally H, et al. A new lineage nomenclature to aid genomic surveillance of dengue virus. PLoS Biol. 2024;22:e3002834. 10.1371/journal.pbio.3002834 39283942 PMC11426435

[6] Holmes EC, Twiddy SS. The origin, emergence and evolutionary genetics of dengue virus. Infect Genet Evol. 2003;3:19–28. 10.1016/S1567-1348(03)00004-2 12797969

[7] Katzelnick LC, Gresh L, Halloran ME, Mercado JC, Kuan G, Gordon A, et al. Antibody-dependent enhancement of severe dengue disease in humans. Science. 2017;358:929–32. 10.1126/science.aan6836 29097492 PMC5858873

[8] Messina JP, Brady OJ, Golding N, Kraemer MUG, Wint GRW, Ray SE, et al. The current and future global distribution and population at risk of dengue. Nat Microbiol. 2019;4:1508–15. 10.1038/s41564-019-0476-8 31182801 PMC6784886

[9] Yu H, Kong Q, Wang J, Qiu X, Wen Y, Yu X, et al. Multiple lineages of dengue virus serotype 2 cosmopolitan genotype caused a local dengue outbreak in Hangzhou, Zhejiang Province, China, in 2017. Sci Rep. 2019;9:7345. 10.1038/s41598-019-43560-5 31089152 PMC6517437

[10] Colón-González FJ, Gibb R, Khan K, Watts A, Lowe R, Brady OJ. Projecting the future incidence and burden of dengue in Southeast Asia. Nat Commun. 2023;14:5439. 10.1038/s41467-023-41017-y 37673859 PMC10482941

[11] Colombia Instituto Nacional de Salud. Identification of the cosmopolitan genotype circulation of the dengue virus in Columbia [in Spanish] [cited 2025 Mar 22]. https://www.ins.gov.co/BibliotecaDigital/comunicado-tecnico-identificacion-de-la-circulacion-genotipo-cosmopolitan-del-virus-del-dengue-2-en-colombia.pdf

[12] Salje H, Lessler J, Maljkovic Berry I, Melendrez MC, Endy T, Kalayanarooj S, et al. Dengue diversity across spatial and temporal scales: Local structure and the effect of host population size. Science. 2017;355:1302–6. 10.1126/science.aaj9384 28336667 PMC5777672

[13] Kiang MV, Santillana M, Chen JT, Onnela JP, Krieger N, Engø-Monsen K, et al. Incorporating human mobility data improves forecasts of Dengue fever in Thailand. Sci Rep. 2021;11:923. 10.1038/s41598-020-79438-0 33441598 PMC7806770

[14] Gubler DJ. Dengue, urbanization and globalization: the unholy trinity of the 21st century. Trop Med Health. 2011;39(Suppl):3–11. 10.2149/tmh.2011-S05 22500131 PMC3317603

[15] Grubaugh ND, Ladner JT, Lemey P, Pybus OG, Rambaut A, Holmes EC, et al. Tracking virus outbreaks in the twenty-first century. Nat Microbiol. 2019;4:10–9. 10.1038/s41564-018-0296-2 30546099 PMC6345516

