# Supplementary material for: Shifting Dynamics of Dengue Virus Serotype 2 and Emergence of Cosmopolitan Genotype, Costa Rica, 2024
Source: Emerg Infect Dis. 2025 Nov;31(11):2153–8. doi: 10.3201/eid3111.250746 (PMC12704521; doi:10.3201/eid3111.250746)
Supplement: Appendix 1 — Additional information shifting dynamics of dengue virus serotype DENV-2 and emergence of cosmopolitan genotype, Costa Rica, 2024. [file 25-0746-Techapp-s1.pdf]

Article DOI: https://doi.org/10.3201/eid3111.250746

EID cannot ensure accessibility for supplementary materials supplied by authors. Readers who have difficulty accessing supplementary content should contact the authors for assistance.

Shifting Dynamics of Dengue Virus Serotype 2 and Emergence of Cosmopolitan Genotype, Costa Rica, 2024

Appendix 1

Material and Methods

Ethics Statement

The Pan American Health Organization Ethics Review Committee (PAHOERC) reviewed and approved this project (Ref. No. PAHO-2024–08–0029). The samples used in this study were analyzed as part of the routine epidemiologic surveillance of arboviruses at the Virology National Reference Center (CNRV, in Spanish) at Inciensa, which is the official laboratory of the Costa Rican Ministry of Health.

Sample Collection and Whole-Genome Sequencing

Samples were collected from patients exhibiting clinical symptoms consistent with dengue virus infection. Nucleic acid extraction was performed using the QIAamp Viral RNA Mini Kit (Qiagen). Subsequently, the extracted RNA was subjected to real-time reverse transcription PCR (RT-qPCR) targeting DENV serotypes 1–4, as previously described (*1*). Samples that tested positive (n = 133) and presented a cycle threshold (Ct) value of ≤30 were selected for whole-genome amplification using the CDC Next Generation Sequencing Protocol for DENV 1–4 with the Illumina MiSeq platform (*2*). This protocol, developed by the CDC, was transferred to the Arbovirus Diagnosis Laboratory Network of the Americas (RELDA) through the VIGENDA program coordinated by the Pan-American Health Organization (PAHO). Library preparation was carried out using the COVIDseq Kit (Illumina, San Diego, USA), originally developed for SARS-CoV-2 genomic studies but subsequently adapted for other viral targets (*3*,*4*). Sequencing was then conducted on the Illumina MiSeq platform (Illumina, San Diego,

USA), following the manufacturer's recommendations, using V2 cartridges with a 2 × 150 cycles paired-end run. Adaptor trimming and quality filtering of the raw reads were performed using Trim-Galore v0.6.5 (*5*) with default parameters for Illumina paired-end reads. Processed reads were mapped to the reference genome using BWA-MEM v0.7.17–4 (*6*) with default settings. The reference genome used for alignment was the serotype-specific DENV2_Reference_Genome_PR1994, provided by the CDC's Dengue Branch in Puerto Rico. Primer sequences were removed using iVar v1.3.1 (*7*). Consensus sequences were then generated from the aligned reads using Samtools v1.10–13 (*8*) and iVar consensus v1.3.1, applying a minimum coverage depth threshold of 10 reads and the default frequency threshold for consensus base calling. Initial genotyping was performed using the Flavivirus Genotyping Tool (Version 0.1; https://www.rivm.nl/mpf/typingtool/flavivirus/). Subsequently, lineage classification was conducted using either the Genome Detective Dengue Typing Tool or Nextclade (*9*,*10*), employing the DENV-2 reference dataset for lineage determination.

Phylogenetic and Phylodynamic Inferences

We constructed phylogenetic trees to investigate the evolutionary relationship of sequenced DENV2 genomes from Costa Rica (DENV2-III n = 110 and DENV2-II n = 23) in comparison to globally sampled sequences (n = 654 and n = 2213 respectively). Sequence alignment was performed using MAFFT (*11*) and manually curated in AliView (*12*). Preliminary maximum likelihood phylogenies were reconstructed using IQ-TREE 2 under the HKY+G4 substitution model (*13*). Time-scaled phylogenies were inferred with TreeTime (*14*), while Bayesian phylogenetic analyses were conducted using BEAST (*15*). To ensure the robustness of the temporal framework, TempEst (*16*) was employed to assess the presence of a temporal signal. For Bayesian inference, we implemented a rigorous model selection approach, using both path-sampling (PS) and stepping-stone (SS) methods to identify the most appropriate molecular clock model (*17*). The uncorrelated relaxed molecular clock was selected for all datasets based on marginal likelihood estimation, in combination with the codon-based SRD06 nt substitution model and the Bayesian Skyline coalescent model. To reconstruct the geographic dissemination of the identified 2022–2023 transmission clade, we employed a relaxed random walk diffusion model (*18*,*19*), which accommodates heterogeneity in dispersal rates among branches, incorporating a Cauchy distribution and a jitter window site of 0.01 (*20*). Each sequence was georeferenced with latitude and longitude coordinates to enable spatiotemporal analyses.

Bayesian phylogenetic inference was performed using BEAST v1.10.4, with two independent Markov Chain Monte Carlo chains (MCMC) runs of 50 million interactions, sampling every 10,000 steps. Convergence was assessed in Tracer, ensuring effective sample size (ESS) >200 for all key parameters. Maximum clade credibility (MCC) trees were summarized using TreeAnnotator after discarding the initial 10% os samples as burn-in. Finally, we employed the R package 'seraphim' version 1.0 (*21*) to extract and visualize spatiotemporal patterns embedded within the posterior tree distribution.

Eco-epidemiologic modeling

The epidemiologic data on weekly confirmed cases of DENV in Costa Rica, from 2013 to 2024, were obtained from the CNRV and aggregated at a monthly level. We calculated the theoretical climate-based transmission potential of the dengue virus using the following mathematical expression (index P), where u stands for humidity and t for temperature:

$$P_{(u,t)} = \frac{{a^{v}_{(u)}}^{2}\,\phi^{v\to h}_{(t)}\,\phi^{h\to v}_{(t)}\,\gamma^{v}_{(t)}\,\gamma^{h}}{\mu^{v}_{(u,t)}(\sigma^{h}+\mu^{h})(\gamma^{h}+\mu^{v}_{(u,t)})} \; (22)$$

Briefly, the index uses mathematical expressions of empirically demonstrated relationships between DENV and *Ae. aegypti* traits and meteorological variables. Climate-dependent traits include the extrinsic incubation period ($\gamma^{v}_{(t)}$), adult mosquito lifespan ($\mu^{v}_{(u,t)}$), adult mosquito biting rate ($a^{v}_{(u)}$transmission probability per mosquito bite from infected human to susceptible mosquito ($\phi^{h\to v}_{(t)}$) and from infected mosquito to susceptible human ($\phi^{v\to h}_{(t)}$). Traits that are climate-independent include intrinsic incubation period (γ^{h}), human lifespan (μ^{h}) and human infectious period (σ^{h}), which are calibrated from published reports. Full methodological details, technical validation and parameterization of Index P can be found in Nakase et al (*22*). Monthly climate data for Costa Rica was obtained from Copernicus.eu satellite climate data (*23*).

References

1. Santiago GA, Vergne E, Quiles Y, Cosme J, Vazquez J, Medina JF, et al. Analytical and clinical performance of the CDC real time RT-PCR assay for detection and typing of dengue virus. PLoS Negl Trop Dis. 2013;7:e2311. PubMed https://doi.org/10.1371/journal.pntd.0002311

2. Vogels CBF, Hill V, Breban MI, Chaguza C, Paul LM, Sodeinde A, et al. DengueSeq: a pan-serotype whole genome amplicon sequencing protocol for dengue virus. BMC Genomics. 2024;25:433. PubMed https://doi.org/10.1186/s12864-024-10350-x

3. Guimarães NR, Tomé LMR, Lamounier LO, Silva MVF, Lima MT, da Costa AVB, et al. Genomic surveillance of monkeypox virus, Minas Gerais, Brazil, 2022. Emerg Infect Dis. 2023;29:1270–3. PubMed https://doi.org/10.3201/eid2906.230113

4. Chen NFG, Chaguza C, Gagne L, Doucette M, Smole S, Buzby E, et al. Development of an amplicon-based sequencing approach in response to the global emergence of mpox. PLoS Biol. 2023;21:e3002151. PubMed https://doi.org/10.1371/journal.pbio.3002151

5. Krueger F. Trim Galore [cited 2025 Mar 22]. https://www.bioinformatics.babraham.ac.uk/projects/trim_galore/

6. Li H, Durbin R. Fast and accurate short read alignment with Burrows-Wheeler transform. Bioinformatics. 2009;25:1754–60. PubMed https://doi.org/10.1093/bioinformatics/btp324

7. Grubaugh ND, Gangavarapu K, Quick J, Matteson NL, De Jesus JG, Main BJ, et al. An amplicon-based sequencing framework for accurately measuring intrahost virus diversity using PrimalSeq and iVar. Genome Biol. 2019;20:8. PubMed https://doi.org/10.1186/s13059-018-1618-7

8. Danecek P, Bonfield JK, Liddle J, Marshall J, Ohan V, Pollard MO, et al. Twelve years of SAMtools and BCFtools. Gigascience. 2021;10:giab008. PubMed https://doi.org/10.1093/gigascience/giab008

9. Vilsker M, Moosa Y, Nooij S, Fonseca V, Ghysens Y, Dumon K, et al. Genome Detective: an automated system for virus identification from high-throughput sequencing data. Bioinformatics. 2019;35:871–3. PubMed https://doi.org/10.1093/bioinformatics/bty695

10. Aksamentov I, Roemer C, Hodcroft EB, Neher RA. Nextclade: clade assignment, mutation calling and quality control for viral genomes. J Open Source Softw. 2021;6:3773. https://doi.org/10.21105/joss.03773

11. Katoh K, Rozewicki J, Yamada KD. MAFFT online service: multiple sequence alignment, interactive sequence choice and visualization. Brief Bioinform. 2019;20:1160–6. PubMed https://doi.org/10.1093/bib/bbx108

12. Larsson A. AliView: a fast and lightweight alignment viewer and editor for large datasets. Bioinformatics. 2014;30:3276–8. PubMed https://doi.org/10.1093/bioinformatics/btu531

13. Nguyen L-T, Schmidt HA, von Haeseler A, Minh BQ. IQ-TREE: a fast and effective stochastic algorithm for estimating maximum-likelihood phylogenies. Mol Biol Evol. 2015;32:268–74. PubMed https://doi.org/10.1093/molbev/msu300

14. Sagulenko P, Puller V, Neher RA. TreeTime: maximum-likelihood phylodynamic analysis. Virus Evol. 2018;4:vex042. PubMed https://doi.org/10.1093/ve/vex042

15. Suchard MA, Lemey P, Baele G, Ayres DL, Drummond AJ, Rambaut A. Bayesian phylogenetic and phylodynamic data integration using BEAST 1.10. Virus Evol. 2018;4:vey016. PubMed https://doi.org/10.1093/ve/vey016

16. Rambaut A, Lam TT, Max Carvalho L, Pybus OG. Exploring the temporal structure of heterochronous sequences using TempEst (formerly Path-O-Gen). Virus Evol. 2016;2:vew007. PubMed https://doi.org/10.1093/ve/vew007

17. Baele G, Li WL, Drummond AJ, Suchard MA, Lemey P. Accurate model selection of relaxed molecular clocks in bayesian phylogenetics. Mol Biol Evol. 2013;30:239–43. PubMed https://doi.org/10.1093/molbev/mss243

18. Lemey P, Rambaut A, Welch JJ, Suchard MA. Phylogeography takes a relaxed random walk in continuous space and time. Mol Biol Evol. 2010;27:1877–85. PubMed https://doi.org/10.1093/molbev/msq067

19. Pybus OG, Suchard MA, Lemey P, Bernardin FJ, Rambaut A, Crawford FW, et al. Unifying the spatial epidemiology and molecular evolution of emerging epidemics. Proc Natl Acad Sci U S A. 2012;109:15066–71. PubMed https://doi.org/10.1073/pnas.1206598109

20. Dellicour S, Gill MS, Faria NR, Rambaut A, Pybus OG, Suchard MA, et al. Relax, keep walking – a practical guide to continuous phylogeographic inference with BEAST. Mol Biol Evol. 2021;38:3486–93. PubMed https://doi.org/10.1093/molbev/msab031

21. Dellicour S, Rose R, Faria NR, Lemey P, Pybus OG. SERAPHIM: studying environmental rasters and phylogenetically informed movements. Bioinformatics. 2016;32:3204–6. PubMed https://doi.org/10.1093/bioinformatics/btw384

22. Nakase T, Giovanetti M, Obolski U, Lourenço J. Population at risk of dengue virus transmission has increased due to coupled climate factors and population growth. Commun Earth Environ. 2024;5:475. https://doi.org/10.1038/s43247-024-01639-6

23. CopernicuPybus OG.s Climate Data Store. Essential climate variables for assessment of climate variability from 1979 to present [cited 2025 Mar 22]. https://cds.climate.copernicus.eu/cdsapp#!/dataset/ecv-for-climate-change

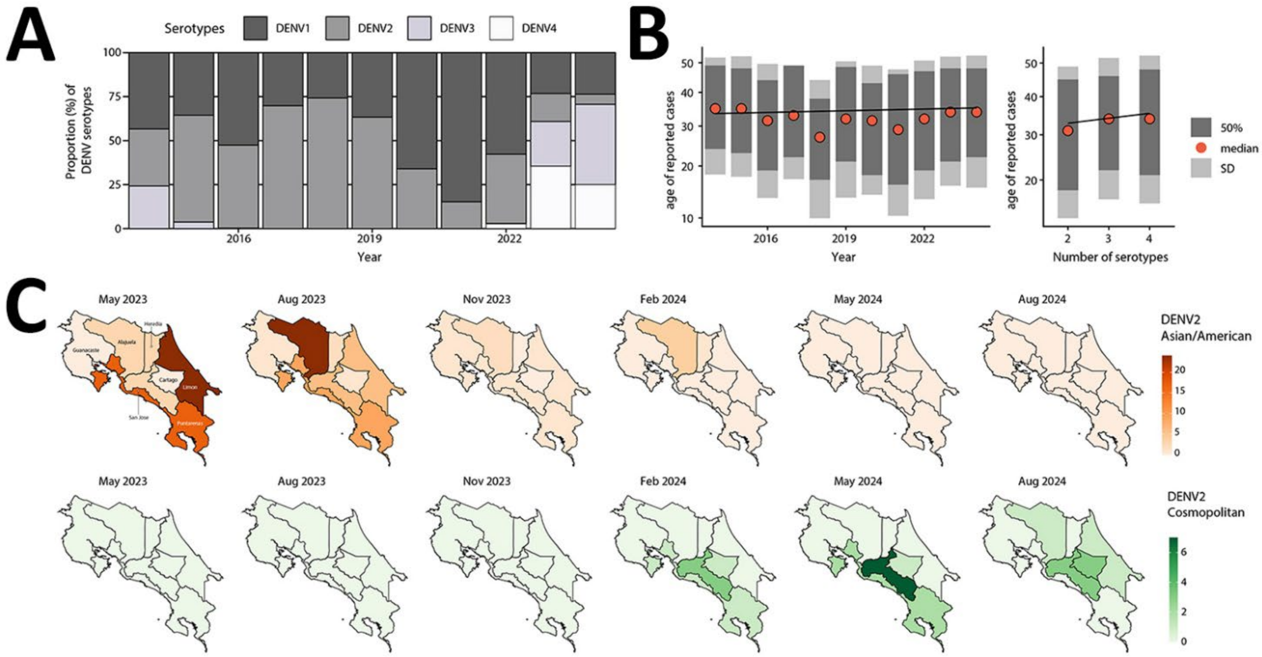

Appendix 1 Figure 1. Dengue serotype dynamics in Costa Rica. A) Proportional distribution of DENV serotypes over time; B) Age of dengue cases over time (left) and by number of circulating serotypes (right). Median (red), interquartile range (dark gray), and standard deviation (light gray) are shown; C) Geographic distribution of DENV2 genotypes in Costa Rica from May 2023 to August 2024.

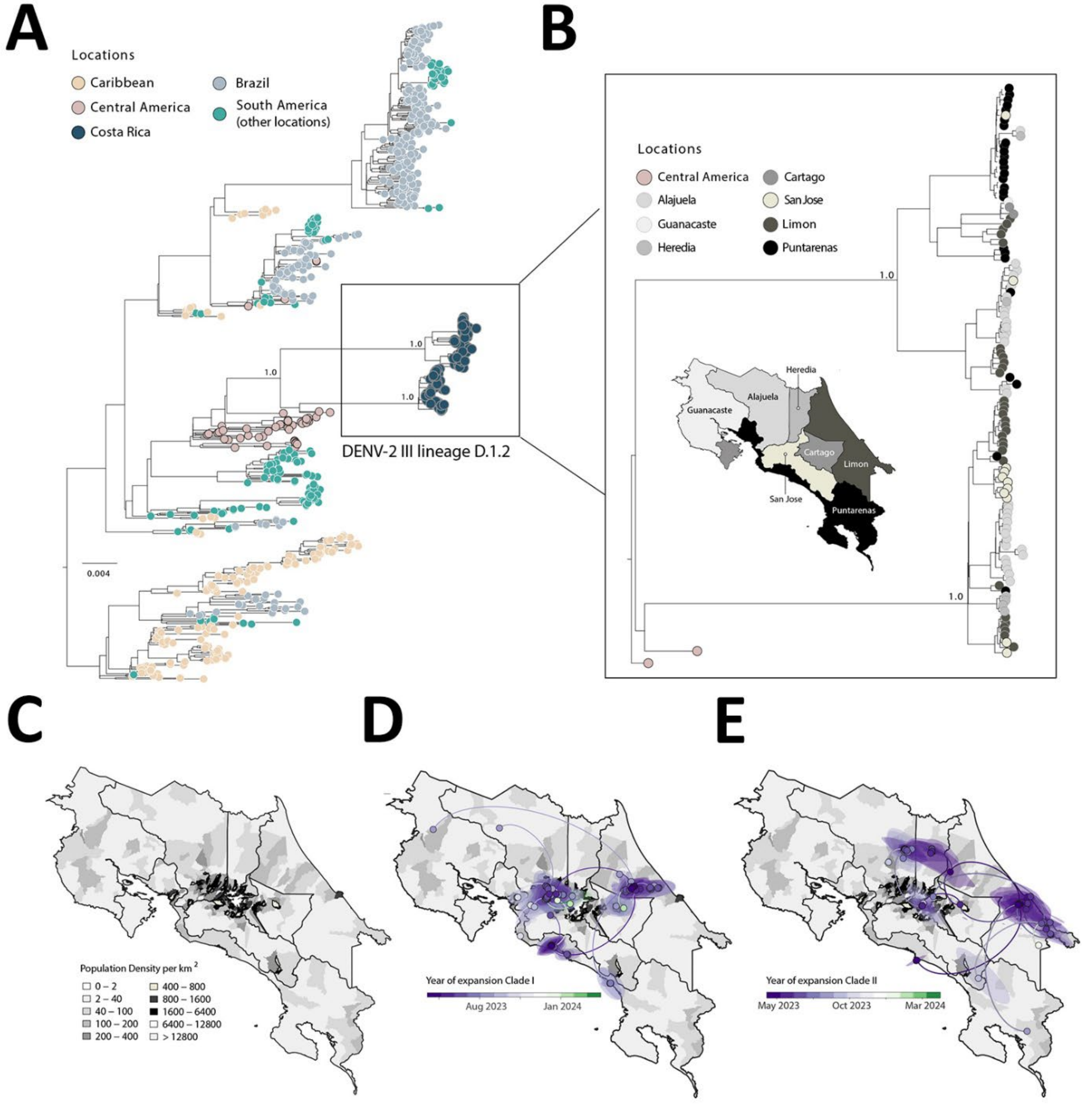

Appendix 1 Figure 2. Evolutionary and spatiotemporal expansion of DENV-2 III in Costa Rica. A) Maximum likelihood (ML) phylogeny of DENV-2 III, showing Costa Rican sequences (blue) within the broader Central and South American clade. Phylogenetic support is indicated at key nodes. B) ML reconstruction of DENV-2 III lineage D.1.2 dispersal within Costa Rica; C) Population density map of Costa Rica, highlighting areas with elevated transmission potential. D, E) Spatiotemporal expansion of DENV-2 III lineage D.1.2, illustrating the progressive spread of two distinct clades—Clade I and II—from urban centers to coastal regions between May 2023 and March 2024.

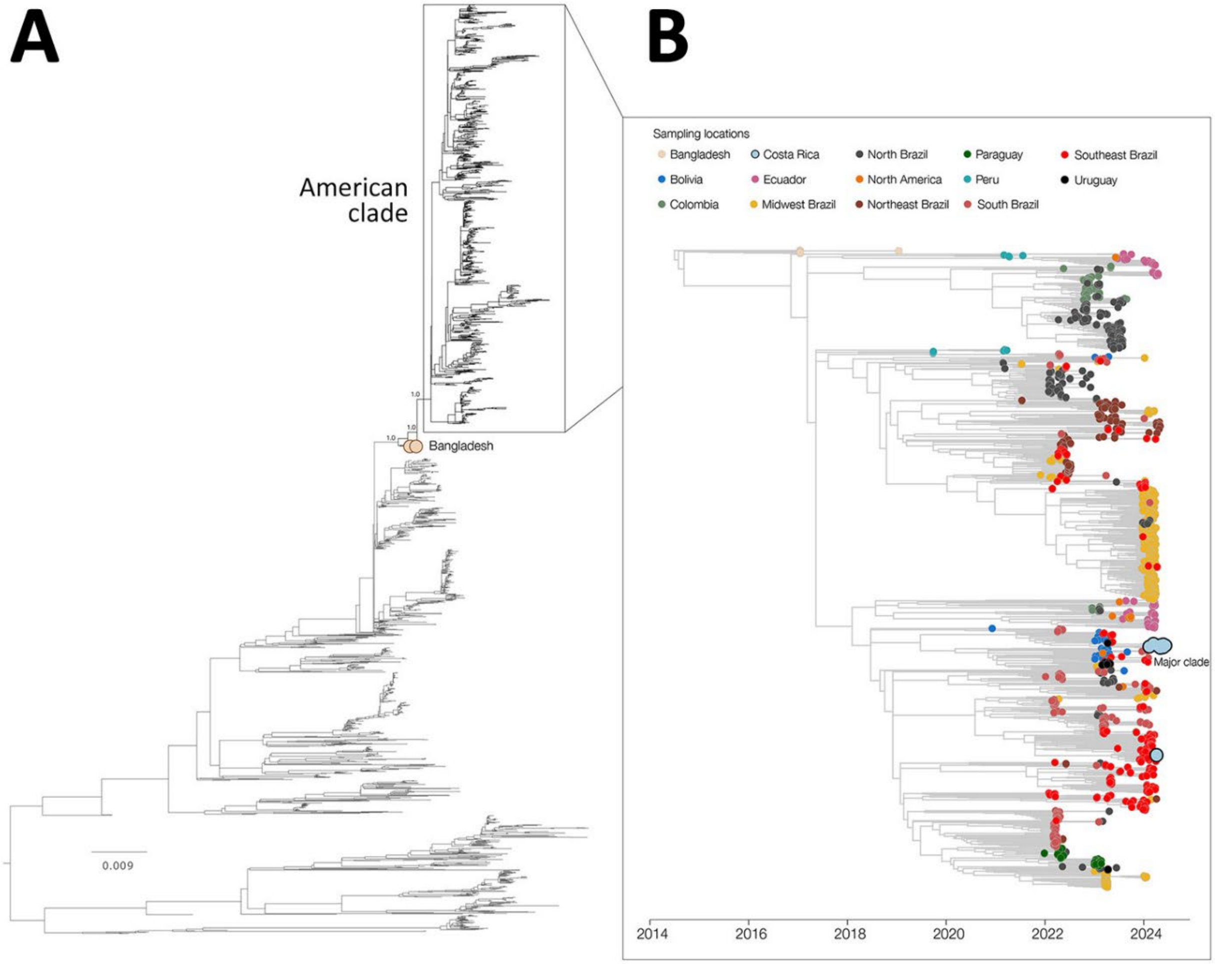

Appendix 1 Figure 3. Emergence and spread of DENV-2 II in Costa Rica. A) Maximum-likelihood phylogeny of DENV-2 II, highlighting the American clade. B) Time-stamped phylogenetic tree, showing 2 independent introductions of this genotype into Costa Rica, including 1 major transmission cluster. Tips are colored according to sampling location. Scale bar indicates nucleotide substitutions per site. DENV, dengue virus.
